# Action Potential Prolongation, β-Adrenergic Stimulation, and Angiotensin II as Co-factors in Sarcoplasmic Reticulum Instability

**DOI:** 10.3389/fphys.2018.01893

**Published:** 2019-01-09

**Authors:** Carlotta Ronchi, Beatrice Badone, Joyce Bernardi, Antonio Zaza

**Affiliations:** Laboratory of Cardiac Cellular Physiology, Department of Biotechnology and Bioscience, University of Milano-Bicocca, Milan, Italy

**Keywords:** action potential duration, angiotensin II, SR stability, arrhythmias, repolarization reserve, β-adrenergic activation

## Abstract

**Introduction:** Increases in action potential duration (APD), genetic or acquired, and arrhythmias are often associated; nonetheless, the relationship between the two phenomena is inconstant, suggesting coexisting factors. β-adrenergic activation increases sarcoplasmic reticulum (SR) Ca^2+^-content; angiotensin II (ATII) may increase cytosolic Ca^2+^ and ROS production, all actions stimulating RyRs opening. Here we test how APD interacts with β-adrenergic and AT-receptor stimulation in facilitating spontaneous Ca^2+^ release events (SCR).

**Methods:** Under “action potential (AP) clamp”, guinea-pig cardiomyocytes (CMs) were driven with long (200 ms), normal (150 ms), and short (100 ms) AP waveforms at a CL of 500 ms; in a subset of CMs, all the 3 waveforms could be tested within the same cell. SCR were detected as inward current transients (I_TI_) following repolarization; I_TI_ incidence and repetition within the same cycle were measured under increasing isoprenaline concentration ([ISO]) alone, or plus 100 nM ATII (30 min incubation+superfusion).

**Results:** I_TI_ incidence and repetition increased with [ISO]; at longer APs the [ISO]-response curve was shifted upward and I_TI_ coupling interval was reduced. ATII increased I_TI_ incidence more at low [ISO] and under normal (as compared to long) APs. Efficacy of AP shortening in suppressing I_TI_ decreased in ATII-treated myocytes and at higher [ISO].

**Conclusions:** AP prolongation sensitized the SR to the destabilizing actions of ISO and ATII. Summation of ISO, ATII and AP duration effects had a “saturating” effect on SCR incidence, thus suggesting convergence on a common factor (RyRs stability) “reset” by the occurrence of spontaneous Ca^2+^ release events.

## Introduction

Prolonged ventricular repolarization, whether caused by myocardial remodeling (Nattel et al., 2007), genetic defects (Schwartz et al., 2001), or drugs (Yang et al., 2001), may reduce myocardial electrical stability. Prolonged action potential duration (APD) may be a reporter of reduced “repolarization reserve,” but it can also be causally linked to arrhythmogenesis through several mechanisms. Along with purely electrophysiological consequences (e.g., increased repolarization heterogeneity) (Winter and Shattock, 2016), APD prolongation affects the sarcolemmal Ca^2+^ influx/efflux balance (Bers, 2001), thus it represents a stress-condition for intracellular Ca^2+^ homeostasis requiring robust compensatory mechanisms. This might be critical under conditions, such as heart failure, in which intracellular Ca^2+^ homeostasis is primarily impaired; indeed, “triggered activity” is common in the failing myocardium (Pogwizd, 1995).

On the other hand, prolonged repolarization *per se* may be insufficient to induce arrhythmias, which might require the concomitance of multiple factors. This may be true also for primarily electrical disorders, such as genetic (Napolitano et al., 2015) and drug-induced (Redfern et al., 2003) repolarization abnormalities. After the early identification of sympathetic activation as a powerful triggering mechanism (Malliani et al., 1980), relatively little attention has been devoted to other biological variables that might theoretically converge with prolonged repolarization in reducing electrical stability. Their identification might pave the way to risk stratification and development of relatively simple preventive measures.

Myocardial and systemic production of angiotensin II (ATII) is increased in response to stress. Beside its well-known role in long-term structural remodeling of the failing heart, ATII has a couple of actions at the cellular level that might acutely compromise the compensations required to cope with prolonged repolarization.

AT-1 receptors (AT1R) stimulation by ATII induces Ca^2+^ release from the sarcoplasmic reticulum (SR) through inositol 1,4,5-trisphospate-receptor channels (IP_3_R) and IP_3_R are functionally coupled to RYR2 in atrial myocytes (Kockskamper et al., 2008; Wullschleger et al., 2017). Although, in ventricular myocytes IP_3_R are less expressed (Garcia and Boehning, 2017), IP_3_R activation (by endothelin 1) has been reported to enhance CICR in rabbit ventricular myocytes (Domeier et al., 2008). Secondly, AT1R stimulation activates production of radical O_2_ species (ROS) by sarcolemmal NADPH-oxydases (Kawai et al., 2017); peroxydation destabilizes RyR2 closed state and is well-known to facilitate spontaneous Ca^2+^ release events (SCR) and related arrhythmias (Prosser et al., 2013).

While an antiarrhythmic effect of ATII antagonism has been reported (Garg et al., 2006), acute proarrhythmia does not stand out as a direct consequence of increased ATII signaling, but what happens if the functional reserve provided by Ca^2+^-homeostatic mechanisms is concomitantly decreased by prolonged repolarization?

Based on the above information, we hypothesize that stimulation of ATII receptors, may facilitate the occurrence of SCR events induced by APD prolongation and β-adrenergic stimulation. The aim of this study was to test such hypothesis.

The practical interest in this question also lies in the availability of widely used drugs limiting ATII cellular effects (AT1R antagonists), which might then be considered as complements in the prevention of ventricular arrhythmias under conditions of prolonged repolarization.

## Materials and Methods

The investigation conforms to the Guide of the Care and Use of Laboratory Animals published by the US National Institute of Health (NIH publication No, 85-23). This study was reviewed and approved by the Animal Care Committee endorsed by University Milano-Bicocca.

### Cell Isolation

Dunkin-Hartley guinea pigs left ventricular cardiomyocytes were isolated by using a retrograde coronary perfusion method previously published (Zaza et al., 1998), with minor modifications. Rod-shaped, Ca^2+^-tolerant myocytes were used within 12 h from dissociation. No measure was taken to dissociate myocytes selectively from either the left or the right ventricle.

### Action Potential Clamp Recordings

Measurements were performed during superfusion with Tyrode's solution at 36.5°C. Pre-recorded guinea-pig action potential (AP) waveforms were applied in V-clamp mode, as previously described (Sala et al., 2017), at a cycle length of 2 Hz. Three AP waveforms (obtained from previous I-clamp recordings under appropriate conditions) with different APD_90_ (APD measured at 90% of repolarization) values were used: 200 ms (Long AP), 150 ms (Normal AP), and 100 ms (Short AP). APD_90_ in the Long AP is 40% longer than in Normal AP, a change proportionally compatible with repolarization abnormalities seen in the clinical setting (Schwartz et al., 1995) (it would simulate a QTc change from e.g., 370–518 ms). According to the experimental protocol, the different AP waveforms were applied to different myocytes (group comparison) or within the same myocyte (internal control). Because of their design, internal control experiments involved application of both AP waveforms in a fixed sequence, thus requiring to exclude the dependence of I_TI_ properties on the sequence itself. This was ruled out in preliminary experiments by applying Long-Short-Long AP sequences within the same myocyte. Characteristics of AP waveforms are summarized in Table S1.

**Figure 1:**
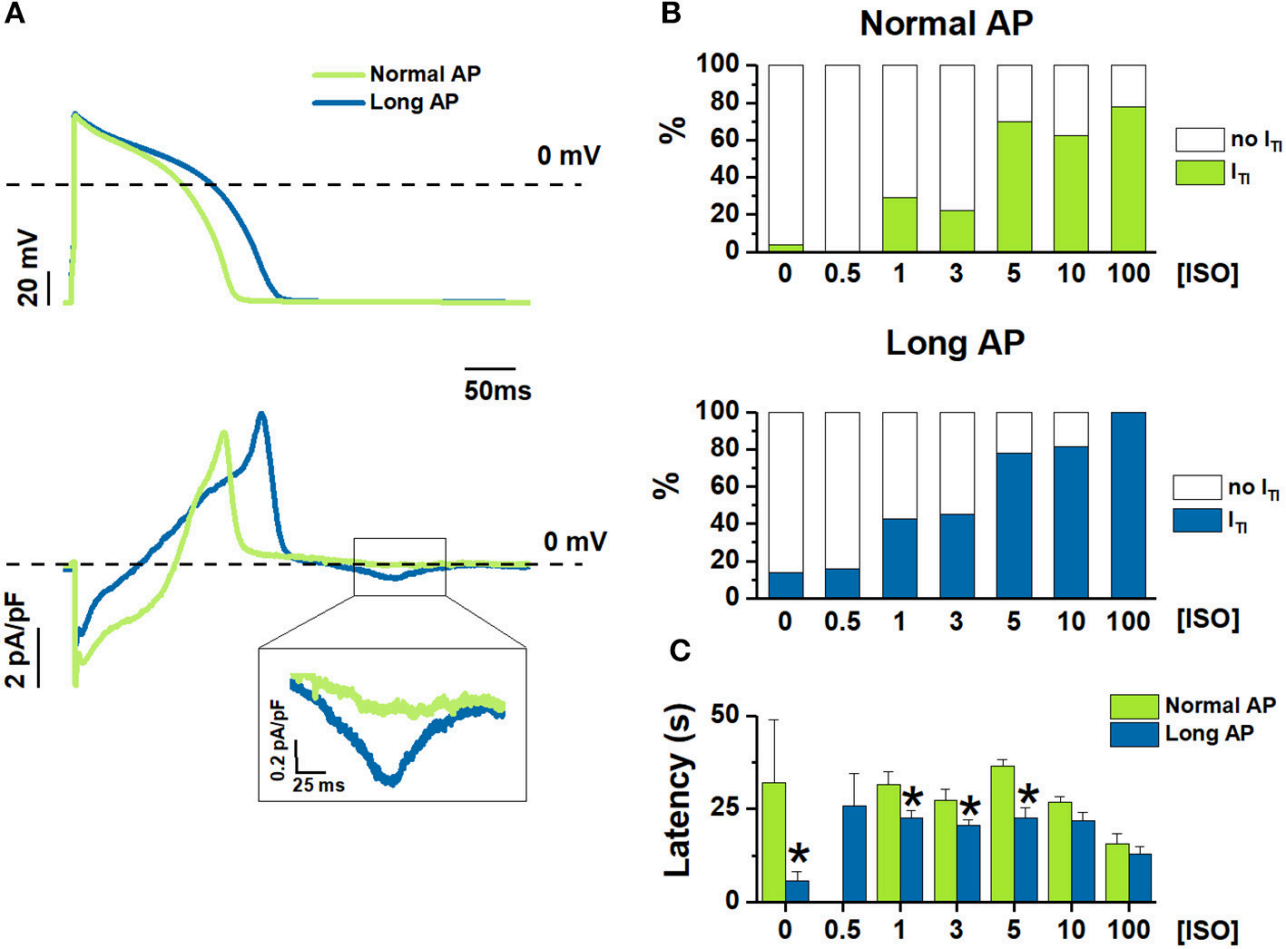
[ISO]-dependency effect on I_TI_ incidence under Normal AP and Long AP. **(A)** Examples of Long AP and Normal AP waveforms used as a command in AP clamp (top) and the corresponding I_m_ (bottom) during 1nM ISO. **(B)** I_TI_ incidence, and **(C)** latency before I_TI_ onset from starting ISO challenge at different ISO concentrations (nM). *N* > 10 for all data points. *P < 0.05 vs. Normal AP.

Total membrane current (I_m_) during the AP-clamp cycle (Figure 1A and Figure S1) was recorded. Because the AP waveforms were not recorded within the same myocyte, I_m_ during the AP was not null as expected under proper “self” AP-clamp conditions (Zaza et al., 1997); nonetheless, changes in the balance between I_m_ inward and outward components could still be used as a gross estimate of interventions effect on systolic currents.

SCR events were detected as the occurrence of diastolic inward current transients (I_TI_), known to result from activation of Na^+^/Ca^2+^ exchange by intracellular Ca^2+^ transients. This method was preferred to direct recording of intracellular Ca^2+^ because free from the contaminating effect of Ca^2+^ buffering by fluorescent Ca^2+^ probes; furthermore, I_m_ is sensitive to sub-sarcolemmal Ca^2+^ changes, which are relevant to membrane electrophysiology but poorly detected by epifluorescence measurements. Based on the baseline variance of diastolic current, I_TI_ events were defined I_TI_ exceeding a threshold of 15 pA; their incidence was defined as the percentage of myocytes in which at least 1 event was observed. Peak I_TI_ amplitude (pA/pF) and I_TI_ integral (Q_TI_, in μC), which reports the charge moved by Na^+^/Ca^2+^ exchange, was used to quantify the magnitude of SCR events. Other parameters considered were I_TI_ coupling interval after the preceding AP (CI in ms) and I_TI_ latency (in sec) after the beginning of ISO infusion.

β-adrenergic stimulation was achieved by exposure to increasing isoprenaline concentrations [ISO]; ATII-receptors were stimulated by 30 min preincubation in 100 nM ATII, which was also added to the superfusate.

Whether the effect of ATII was due to modulation of IP_3_R was tested by (5 min) exposure to 2 μM 2-aminoethyl diphenylborinate (2APB), an antagonist of IP_3_R. Nifedipine 5 μM (NIFE) was used to compare 2APB effect on I_m_ to that of L type Ca^2+^ current (I_CaL_) blockade.

### Experimental Solutions

Tyrode's solution contained (in mM): 154 NaCl, 4 KCl, 2 CaCl_2_, 1 MgCl_2_, 5 HEPES-NaOH, 5.5 D-glucose, adjusted to pH 7.35 with NaOH. Myocytes were patch-clamped with borosilicate glass pipettes containing (mM): K^+^-aspartate 110, KCl 23, MgCl_2_ 3, HEPES KOH 5, EGTA KOH 1, GTP Na^+^-salt 0.4, ATP Na^+^-salt 5, creatine phosphate Na^+^-salt 5, CaCl_2_ 0.4 (calculated free-Ca^2+^ = 10^−7^ M), adjusted to pH 7.3.

ISO and ATII were dissolved in water, NIFE in ethanol, and 2APB in DMSO. When used, compound solvents (ethanol or DMSO) were added to all solutions at the same final concentration, which never exceed 0.1%. All chemicals were purchased from Sigma.

### Statistical Analysis

Student's paired or unpaired *t*-test was applied to compare means of continuous variables (latency, amplitudes etc). Difference between categorical variables (expressed as incidence %) was tested by chi-square (group comparison) or McNemar analysis (internal control) applied to absolute numbers; GLM regression for binomial data (R statistical package) was used to compare vectors of categorical variables (incidence in ISO dose-response curves). Statistical significance was defined as *P* < 0.05. Sample size (n, number of cells) is specified for each experimental condition in the respective figure legend.

Categorical variables are expressed in percentages while the continuous variables are expressed as average ± standard error of the mean.

## Results

### Effect of APD Prolongation on ISO-Induced I_TI_ Events

The experimental protocol was optimized to test the effect of multiple [ISO] in each myocyte; because of limitations inherent to preparation stability, the effect of changing APD on the [ISO] vector had to be tested by comparing myocyte groups. Thus, the incidence of I_TI_ events was compared between myocytes clamped with either the Normal AP or the Long AP (group comparison) at baseline and during exposure to increasing ISO concentrations ([ISO]) (Figure 1).

I_TI_ incidence dose-dependently increased with [ISO] under both AP waveforms (Figure 1B); the Long APD moved the ISO concentration-response curve to significantly higher I_TI_ incidences (*p* < 0.05 at binomial GLM regression) and reduced the threshold concentration for ISO effect (lower vs. upper panel in Figure 1B). As compared to Normal AP, Long AP shortened the time of I_TI_ appearance after the beginning of ISO (or control) solution challenge; however, this effect became smaller as [ISO] was increased (Figure 1C).

### Effect of Adding ATII

**Figure 2:**
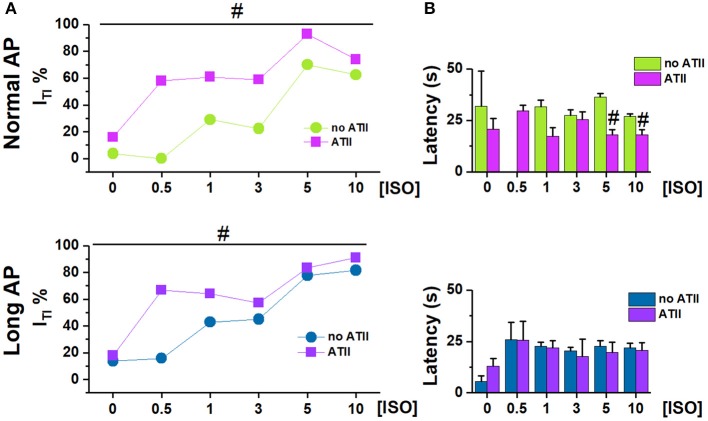
Effect of adding ATII on I_TI_ incidence under Normal AP and Long AP. **(A)** I_TI_ incidence and **(B)** latency at different ISO concentrations (nM) in untreated (noATII) and ATII- treated (ATII) CMs. *N* > 20 for all groups. #*p* < 0.05 vs. ISO group.

The above experiments were repeated in cells treated with 100 nM ATII (ATII) and compared with untreated cells (no ATII) (Figure 2).

ATII alone [[ISO] = 0] had a very small effect on I_TI_ incidence under Normal AP (*p* < 0.05) and failed to change I_TI_ significantly under Long AP (Figure 2A). Within each AP waveform, ATII moved the ISO concentration-response curve to significantly higher I_TI_ incidences. ATII effect was present with both AP waveforms, (*p* < 0.05 at binomial GLM regression), but it was smaller and limited to low [ISO] during the Long AP (Figure 2A). ATII tended to shorten the time of I_TI_ appearance (latency) after the beginning of ISO challenge, but did so only under the Normal AP (Figure 2B).

### Effect of APD Changes on I_TI_ Occurrence and Properties

**Figure 3:**
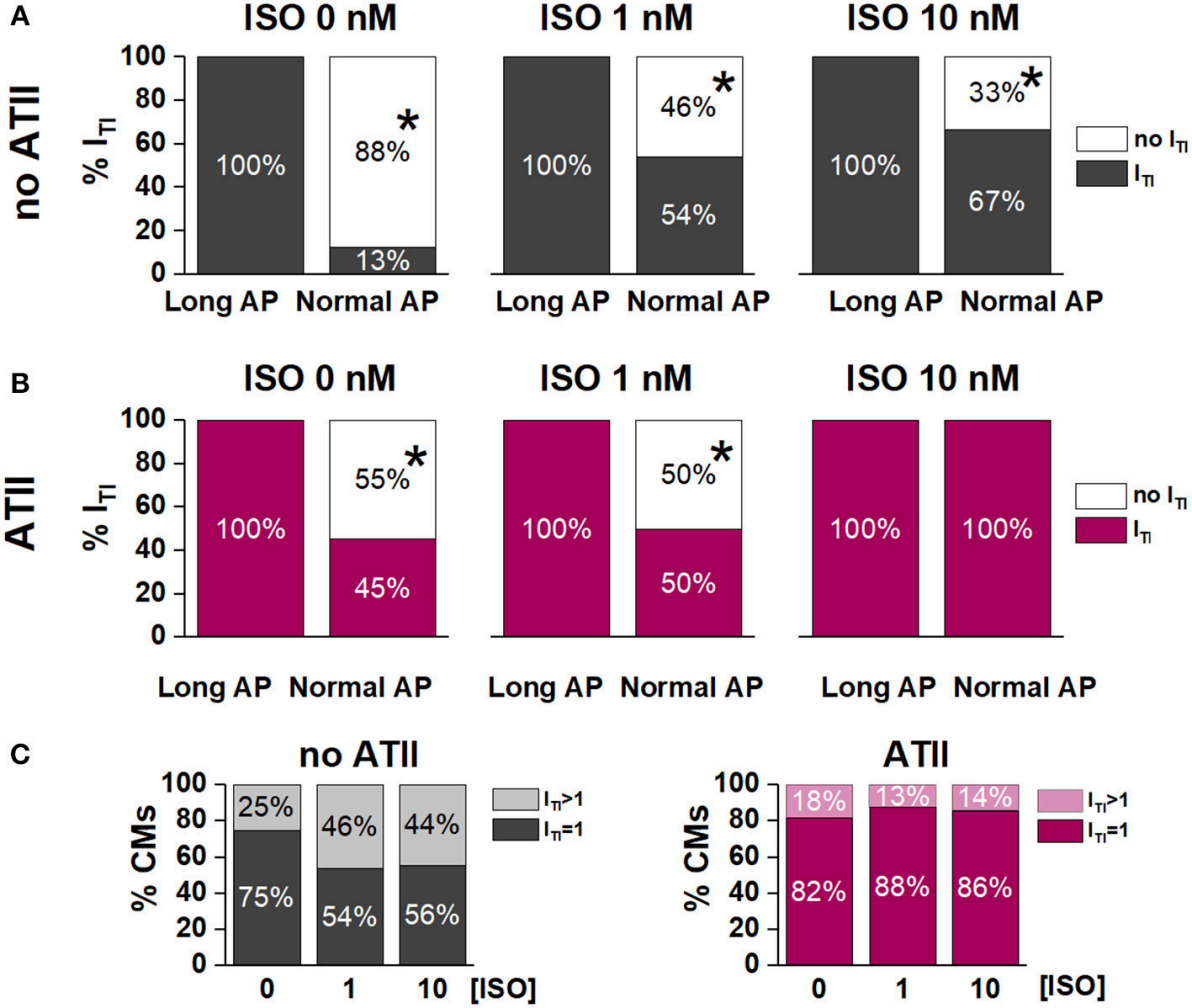
Effect of APD shortening on I_TI_ incidence within the same cardiomyocyte in the absence (no ATII) **(A)** or presence of ATII (ATII) **(B)**. **(C)** Percent of cardiomyocytes with 1 I_TI_ event or >1 I_TI_ events in the absence (no ATII) or presence (ATII) of ATII (Long AP; 0, 1,10 nM ISO). CMs: cardiomyocytes. *N* > 8 for all groups. *p < 0.05 vs. Long AP.

**Figure 4:**
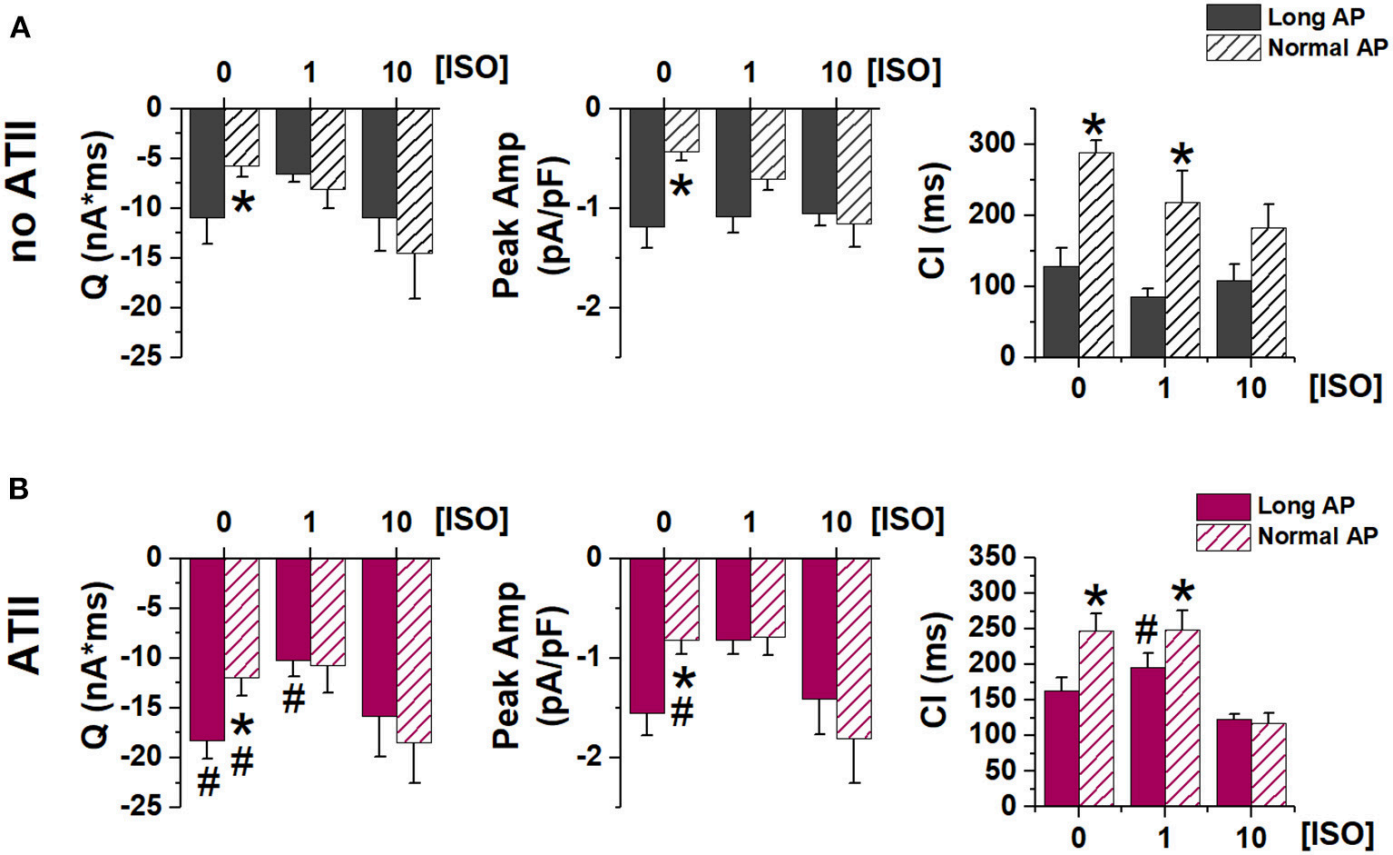
Effect of APD shortening on I_TI_ properties. Statistics (mean ± S.E.) of I_TI_ charge (Q), peak I_TI_ amplitude (Peak Amp) and coupling interval (CI) **(A)** in the absence of ATII (no ATII); **(B)** in the presence of ATII (ATII). *N* > 8 for all groups. *p < 0.05 vs. Long AP; #*p* < 0.05 vs. no ATII CMs.

This set of experiments was designed to test whether APD shortening would effectively modify I_TI_ events induced by receptor stimulation. To this end, each myocyte in which I_TI_ events occurred during the Long AP (incidence = 100% by default) was subsequently clamped with the Normal AP (internal control). This was repeated in myocyte groups, each exposed to a different [ISO] alone, or in the presence of ATII (Figures 3, 4). I_TI_ incidence and properties were measured.

Without ATII, (Figure 3A), switching from Long AP to Normal AP caused I_TI_ to cease within several beats in 88% of the cells in basal conditions, in 46% under 1 nM ISO and in 33% under 10 nM ISO (*p* < 0.05 for ΔAPD_90_ differences along the [ISO] vector). In ATII-treated myocytes, APD shortening terminated I_TI_ in 55% of cells under basal conditions and termination rate decreased with increasing [ISO] (Figure 3B). The effect of APD shortening on I_TI_ incidence was not significantly modified by ATII.

In the absence of ATII, occurrence of two I_TI_ events within the same cycle was not significantly affected by ISO. Surprisingly, ATII did not increase the occurrence of double I_TI_ events and, if anything, tended to reduce it (Figure 3C).

Similarly, I_TI_ properties were affected by APD shortening (in the direction of smaller SCR with greater latency) only in the absence of β-adrenergic stimulation. ATII increased I_TI_ magnitude (Q_TI_ and peak amplitude), but the effect of APD shortening was preserved (Figure 4).

**Figure 5:**
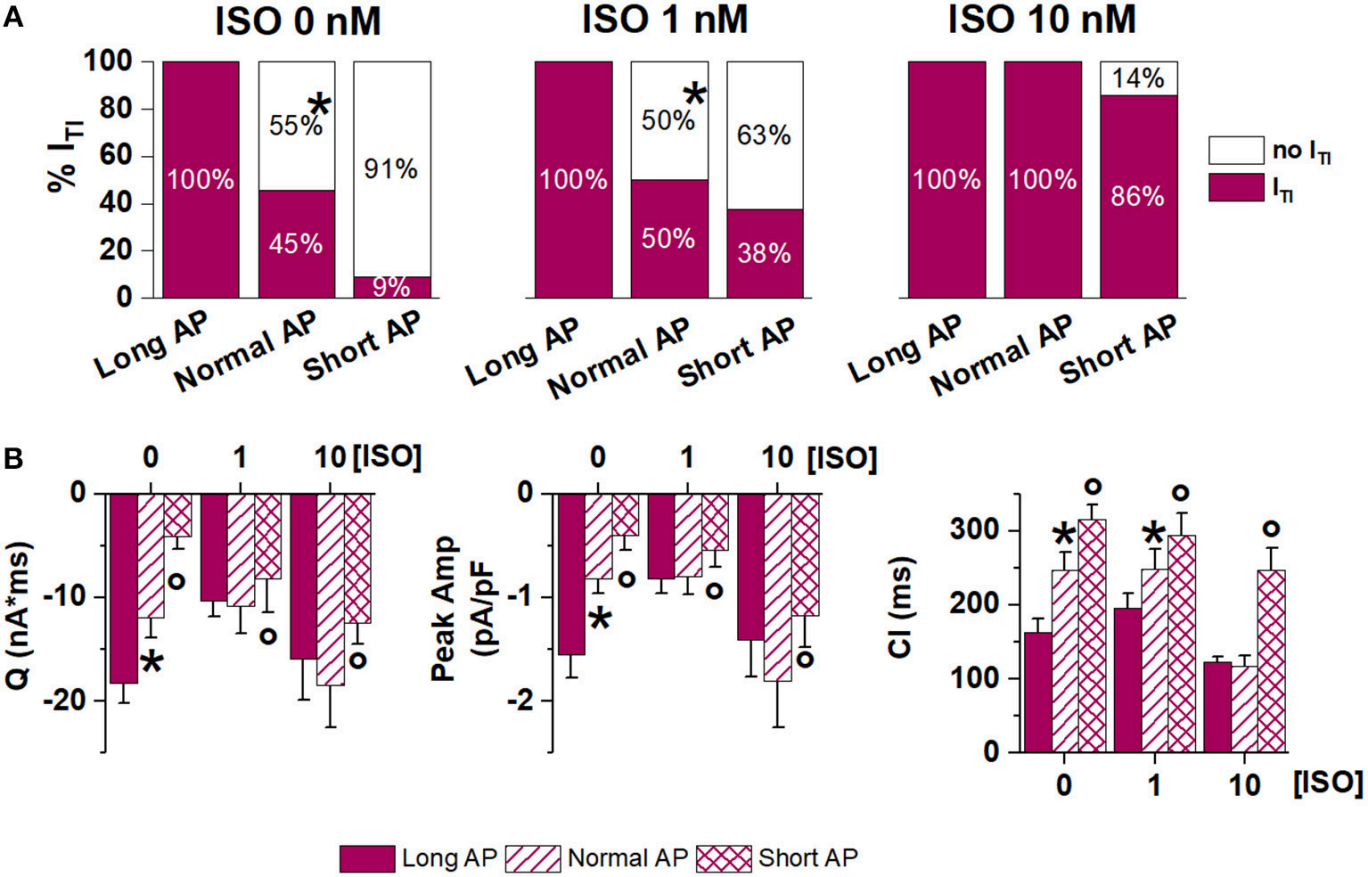
Effect of APD shortening within the same ATII-treated cardiomyocyte (Long AP, Normal AP and Short AP waveforms). **(A)** I_TI_ incidence in basal condition (ISO 0) and in 1 nM and 10 nM ISO. **(B)** statistics (mean ± S.E.) of I_TI_ charge (Q), peak I_TI_ amplitude (Peak Amp) and coupling interval (CI). *N* > 8 for all groups *p < 0.05 vs. Long AP; °*p* < 0.05 vs. Normal AP.

To test whether APD affected I_TI_ occurrence only if increased above its normal value, in a subset of myocytes the Short AP waveform was also tested (again by internal control) (Figure 5). I_TI_ incidence and properties were incrementally modified by AP waveforms of shorter duration, suggesting that SR stability depends on APD in a continuous fashion. High [ISO] minimized the effect of APD shortening.

Overall, internal control experiments confirmed that APD shortening may counter the arrhythmogenic effect of receptor stimulation, but its impact was reduced at high levels of neurohumoral stimulation.

### IP_3_R Contribution to ATII Effect

**Figure 6:**
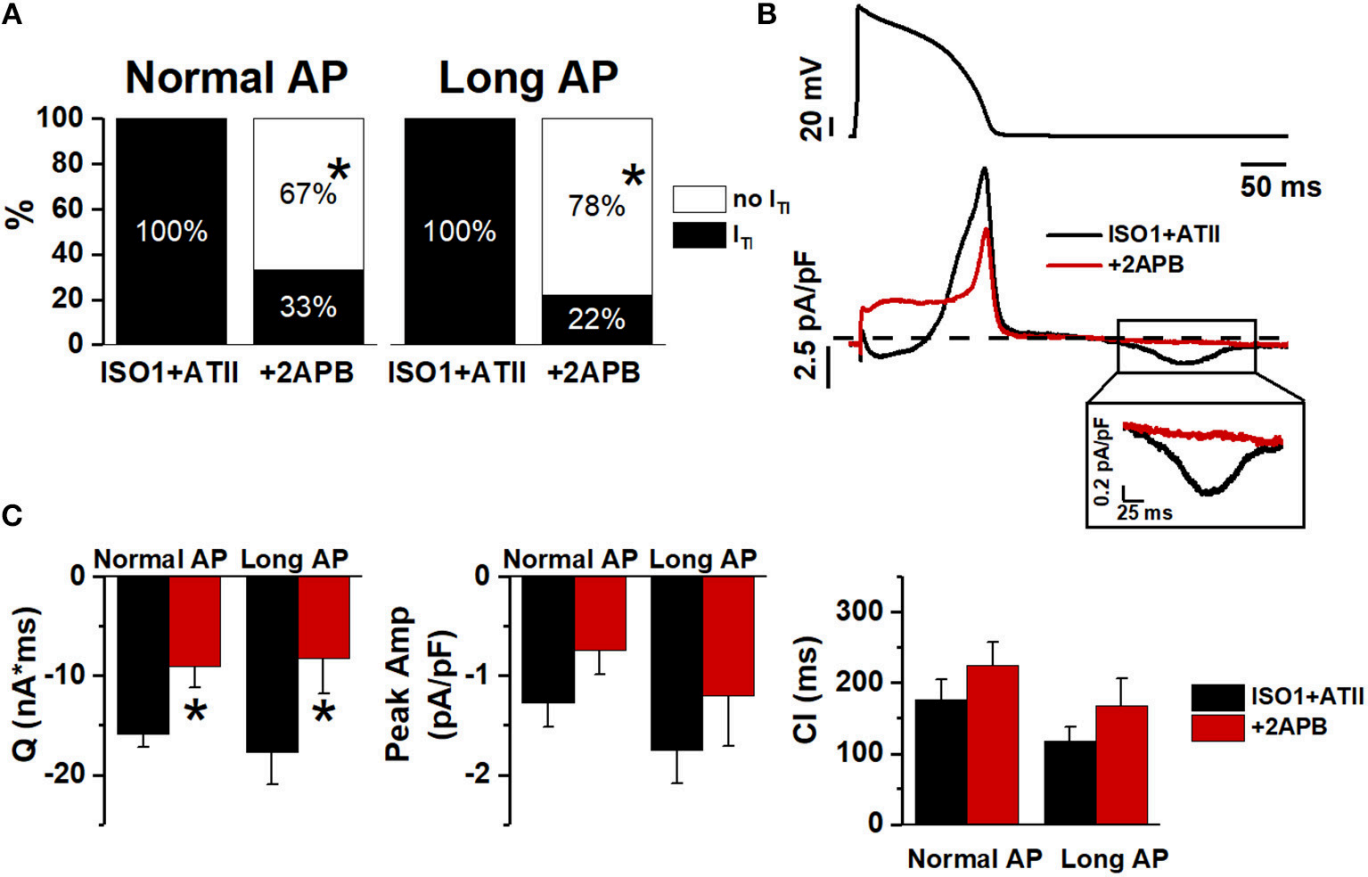
Effect of 2APB on I_TI_ induced by ISO+ATII. **(A)** I_TI_ incidence in ISO1+ATII alone and with 2APB. **(B)** Examples of Normal AP (top) and I_m_ corresponding (bottom). **(C)** statistic (mean ± S.E.) of I_TI_ charge (Q); peak I_TI_ amplitude (Peak Amp) and coupling interval (CI). *N* = 9 for both groups. *p > 0.05 vs. ISO1+ATII.

The contribution of IP_3_ signaling to I_TI_ facilitation by ATII was investigated by blocking IP_3_R (by 2APB) (Figure 6). To this end I_TI_ incidence was measured, during Normal and Long APs, in the presence of 1 nM or 10 nM ISO + ATII only and after adding 2APB to the superfusate.

With 1 nM ISO, 2APB similarly reduced I_TI_ incidence during Normal APD and Long APD (Figure 6A); the same was true for I_TI_ charge (Q) (Figure 6C). When [ISO] was increased to 10 nM 2APB still reduced I_TI_ incidence during Normal AP (by 60%) but had a much smaller effect during the Long AP (Figure S2).

This would suggest that IP_3_R stimulation significantly contributed to ATII-induced increment in SCR events; however, a substantial outward shift of I_m_ during the AP (Figure 6B) opens the possibility of decreased Ca^2+^ influx as the mechanism for I_TI_ suppression. To test the hypothesis of direct I_CaL_ blockade by 2APB, we compared the kinetics of its action on I_m_ during the AP plateau to that of a saturating concentration of NIFE (Figure S3). The much slower kinetics of 2APB argues against direct I_CaL_ blockade by 2APB.

## Discussion

The main findings of the present study were: ISO concentration-dependently induced SCR events and APD prolongation enhanced ISO effect; ATII increased SCR incidence preferentially when it was still far from 100%, i.e., with normal APD and in the lower range of [ISO].

While APD prolongation typically facilitates EADs through a mechanism at least partially independent of SCR (January and Riddle, 1989; Song et al., 2015), its association with SCR is less straightforward. An increase in intracellular Ca^2+^ content (both on the cytosolic and luminal sides of SR membrane) facilitates SCR (Egdell et al., 2000; Venetucci et al., 2008). Because of net charge transport by the Na^+^/Ca^2+^ exchanger, Ca^2+^ extrusion from the cell is favored at diastolic potentials; thus, assuming a constant cycle length (CL), APD prolongation can increase intracellular Ca^2+^ content simply by decreasing the proportion of the cycle occupied by diastole (Bers, 2001). Accordingly, modeling studies indicate that the amplitude and prematurity of DADs is expectedly related to APD because of APD-dependency of SR Ca^2+^ load (Song et al., 2015). Nonetheless, blockade of a repolarizing current (I_Ks_) in canine myocytes prolonged ventricular APD (by ~15%) but failed to induce DADs unless associated with β-adrenergic stimulation (Burashnikov and Antzelevitch, 2000). In the present study, APD prolongation (by 40%) alone slightly increased I_TI_ incidence also in the absence of ISO; this effect was smaller than that previously reported in rabbit myocytes using more pronounced APD prolongation (Wu et al., 1999). APD prolongation had a much larger effect in the presence of very low (nanomolar) ISO concentrations (Figure 1). This suggests that APD prolongation by amounts more relevant to the clinical setting may be relatively inconsequential on its own but becomes a powerful co-factor in the induction of SCR by adrenergic stimulation.

In the present experiments, APD was changed at a constant cycle length, thus reciprocally affecting diastolic interval. This implies that membrane “duty cycle” (the ratio between systolic and diastolic times), rather than APD itself, was considered. This approach was motivated by the consideration that repolarization abnormalities are defined as changes of the rate-corrected QT interval, which implies that they are not accompanied by a commensurate change in cycle length. Furthermore, from a mechanistic standpoint, the sarcolemmal Ca^2+^ flux balance depends on membrane duty cycle, rather than on APD alone (Bers, 2001). Nonetheless, it is fair to stress that the present data may not describe the effect of changes in APD alone (i.e., with constant diastolic interval).

In the presence of normal APD, ATII significantly facilitated I_TI_ induction at all levels of β-adrenergic stimulation (Figure 2). ATII has been reported to exert “enhanced antagonism” of I_CaL_ activation by β1-AR, reflecting inhibition of pre-activated adenylate cyclase (Ai et al., 1998). Thus, I_TI_ facilitation by ATII in the context of β1-AR activation may look contradictory. However, it should be noted that, in the present experiments, I_TI_ facilitation by ATII prevailed at low ISO concentrations, which failed to induce I_TI_ events on their own (Figure 2). Overall, this may suggest that ATII facilitated I_TI_ events by mechanisms other than those triggered by β1-AR activation, but converging with them to impair SR stability.

ATII activates membrane phospholipase C (PLC) resulting in production of IP_3_ and diacylglycerol. SR Ca^2+^ release through IP_3_R channels is central in smooth muscle physiology and significantly contributes to intracellular Ca^2+^ dynamics of atrial myocytes (Li et al., 2005; Wullschleger et al., 2017). Expression of IP_3_R in ventricular myocytes is lower, largely perinuclear and likely devoted to excitation-transcription coupling (Lipp et al., 2000; Wu et al., 2006; Domeier et al., 2008); therefore, their role in intracellular Ca^2+^ dynamics is debated. Nevertheless, IP_3_-dependent enhancement of excitation-contraction coupling has been observed in rabbit ventricular myocytes (Domeier et al., 2008). Functional crosstalk between neighboring IP_3_R and RyR2 channels, caused by local Ca^2+^ diffusion and amounting to direct activation of Ca^2+^ release units, has been demonstrated in atrial myocytes (Wullschleger et al., 2017). In ventricular myocytes (including human ones), enhanced IP_3_R-mediated SR Ca^2+^ leak has been reported not to affect RyR2 function directly, but rather secondary to APD prolongation by the increment in Na^+^/Ca^2+^ exchanger current (I_NCX_) (Signore et al., 2013). Consistent with this interpretation, IP_3_R overexpression in a transgenic mouse model substantially enhanced SR Ca^2+^ leak without affecting the incidence of organized SCR events (Ca^2+^ sparks or puffs) (Blanch and Egger, 2018).

IP_3_R activation likely contributed to SCR facilitation by ATII in the present experiments, as suggested by the effect of 2APB (Figure 6). 2APB also shifted the current balance during the AP in the outward direction, a change compatible with an increased contribution of I_NCX_ during IP_3_R activation (Signore et al., 2013). Direct I_CaL_ blockade by 2APB is unlikely because of the slow kinetics of 2APB effect (Figure 6) and of failure 2APB alone to affect Ca^2+^ dynamics in ventricular myocytes (Peppiatt et al., 2003). The present results suggest that IP_3_R activation, possibly through an increment of cytosolic Ca^2+^, may indeed facilitate Ca^2+^ waves under the conditions generated by APD prolongation and β-adrenergic stimulation.

Albeit not investigated in the present work, activation of ROS production by ATII is a further mechanism potentially contributing to RyR2 destabilization (Prosser et al., 2013).

Seen in the context of previous findings, the present results suggest that, albeit potentially inconsequential on its own, ATII may substantially facilitate SCR induction by β-adrenergic stimulation in ventricular myocytes.

Apparently in contrast with the hypothesis of a facilitatory role of APD prolongation, when APD was prolonged, ATII effect was smaller and limited to very low ISO concentrations (Figure 2). However, this can be viewed as “saturation” of the mechanisms responsible for SCR by APD prolongation (i.e., intracellular Ca^2+^ load) and strong β-adrenergic stimulation. Notably, even at saturation, I_TI_ incidence did not achieve 100% and events per cycle were limited to one or two. Such pattern is expected from convergence of the three co-factors (APD, ISO and ATII) on a common mechanism (RyR2 facilitation) with a “ceiling” effect. The ceiling can be conceivably caused by the SCR itself, which resets SR Ca^2+^ content (Venetucci et al., 2008). Considering that ATII facilitated SCR, we expected it to increase the occurrence of double I_TI_ events within a cycle. The finding that the opposite was true can be tentatively interpreted by considering that ATII increased the magnitude of the first SCR event (Q in Figure 4); by causing larger SR Ca^2+^ depletion, this might reduce the probability of a second SCR event.

The relevance of APD on I_TI_ induction is highlighted by the ability of APD shortening to abrogate I_TI_ once it was induced by either ISO alone or ISO plus ATII (Figure 5). Even when I_TI_ persisted after AP normalization, its charge and amplitude were reduced, indicating that APD may affect the amount of Ca^2+^ released by the SCR and, with it, the likelihood of triggering a propagated beat. In keeping with the convergence of factors, the ability of APD normalization to affect I_TI_ occurrence and properties was reduced at high ISO concentrations and, further, in the presence of ATII.

## Conclusion

From the data obtained it is possible to conclude that ATII may act as a co-factor to facilitate the occurrence of SCR in the presence of AP prolongation and β-adrenergic stimulation.

## Limitations

Among small animals, guinea-pigs have an action potential contour which is closest to the human one; this justifies their adoption as experimental model. However, as compared to canine (and, possibly, human) myocytes, guinea-pig ones do not express I_TO_ (Nakajima et al., 2002) and have a prominent sustained I_CaL_, which likely supports a more positive action potential plateau (Sala et al., 2017). This might exaggerate the impact of changes in APD in facilitating SCR events.

While I_TI_ (measured under V-clamp) is a suitable reporter of SCR, the direct cause of arrhythmogenesis by SCR are DADs. Albeit I_TI_ is the cause of DAD, their features do not necessarily coincide because V-clamp prevents the positive feed-back loop between depolarization and further current activation; moreover, the amplitude of DADs caused by a given I_TI_ amplitude depends on membrane diastolic resistance and, thus, on the currents determining it (mostly I_K1_). While absence of positive feed-back would lead to underestimate DADs amplitude, the effect of the interventions tested on diastolic membrane resistance is unknown.

## Pratical Implications

ATII signaling is part of an homeostatic control system central to physiological adaptations; furthermore, ATII is also synthetized locally (cardiomyocytes, fibroblasts, vascular cells) in response to stress (Varagic and Frohlich, 2002); its production and its receptors (AT1R) are upregulated in cardiac disease (Szczepanska-Sadowska et al., 2018). Therefore, the extent of ATII signaling may well vary among subjects and, within a subject, in response to physiological and pathological states. The present results in cardiomyocytes suggest that ATII signaling might act as a variable co-factor in determining the impact of repolarization abnormalities on SR stability and the resulting arrhythmias. Thus, ATII antagonism might have an antiarrhythmic significance beyond prevention of myocardial remodeling, potentially extended to genetic and drug-induced conditions of prolonged repolarization. Considering that myocardial ATII production may be independent of the angiotensin converting enzyme (ACE) (Varagic and Frohlich, 2002), direct antagonism of AT1 receptors should be considered as the logical intervention.

## Author Contributions

CR planned, performed, analyzed the majority of experiments and wrote the first manuscript draft; BB and JB contributed to part of the experiments and to data analysis. AZ supervised the study and contributed to the manuscript final version.

### Conflict of Interest Statement

The authors declare that the research was conducted in the absence of any commercial or financial relationships that could be construed as a potential conflict of interest.
