# Supplementary material for: Action Potential Prolongation, β-Adrenergic Stimulation, and Angiotensin II as Co-factors in Sarcoplasmic Reticulum Instability
Source: Front Physiol. 2019 Jan 9;9:1893. doi: 10.3389/fphys.2018.01893 (PMC6333690; doi:10.3389/fphys.2018.01893)
Supplement: Supplementary file 1 [file Data_Sheet_1.PDF]

Action potential prolongation, β-adrenergic stimulation and angiotensin II as co-factors in sarcoplasmic reticulum instability

Carlotta Ronchi et al.

SUPPLEMENTAL FIGURES

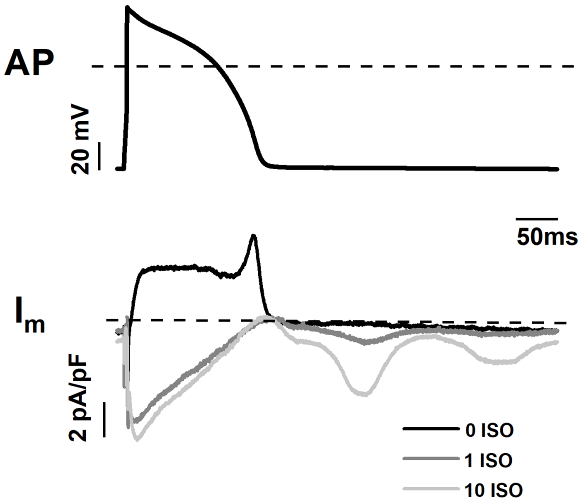

Figure S1: [ISO]-dependency effect on I_{TI} occurrence. Examples of Normal AP waveform used as a command in AP clamp (top) and the corresponding I_m (bottom) in basal condition and under ISO exposure.

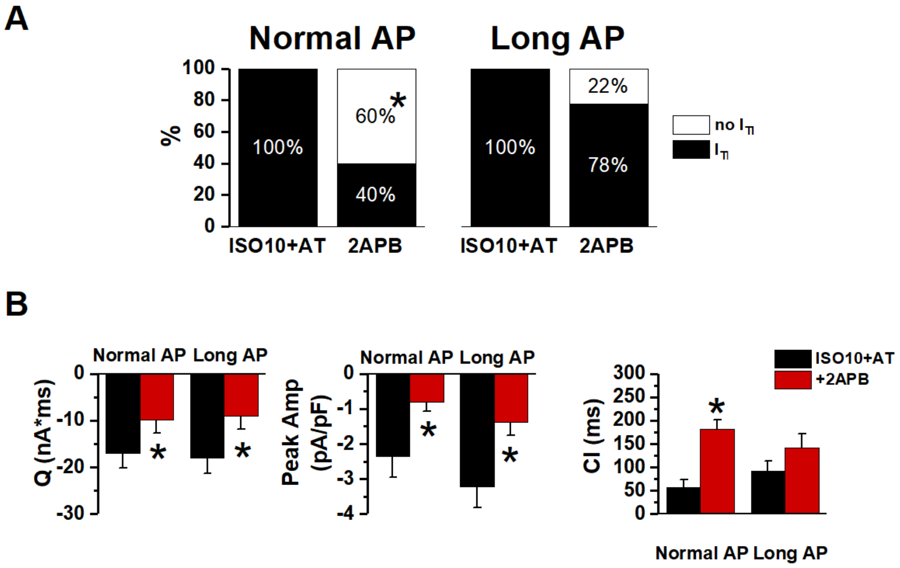

Figure S2: 2APB effect on I_{TI} evocated by ISO10+AT within same CMs. (A) I_{TI} incidence in ISO10+AT alone and with 2APB. **(B)** statistic (mean ± S.E.) of I_{TI} charge (Q); peak ITI amplitude (Peak Amp) and coupling interval (CI). Normal AP N=10, Long AP N=9. * $p > 0.05$ vs ISO10+AT.

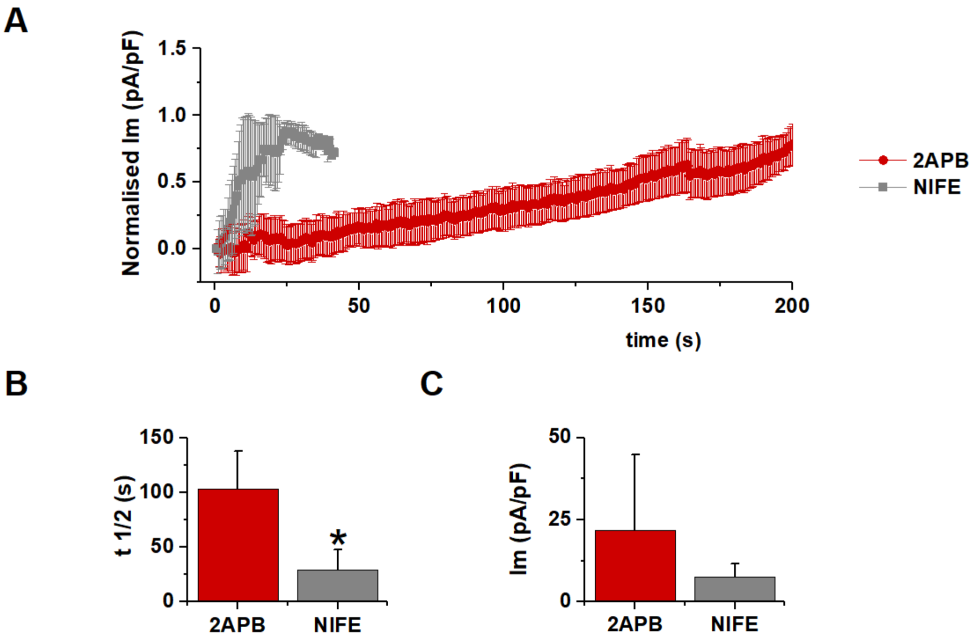

FigureS3: 2APB and NIFE effect on I_m during AP plateu phase under 10nM ISO exposure. (A) Time course of means ± S.E. of normalized I_m values during AP plateau phase in the presence of 2APB (red) or NIFE (grey). **(B)** Time for half-maximal effect of 2APB and NIFE. (C) Maximal effect of 2APB and NIFE on Im. 2APB N=6, NIFE N=5. * $p > 0.05$ vs 2APB

	**Long AP**	**Normal AP**	**Short AP**
**E_{diast} (mV)**	-80.1103	-80.354	-80.5857
**PA (mV)**	47.8344	46.83809	53.33333
**APD_{20} (ms)**	72.6823	61.297	37.41788
**APD_{50} (ms)**	151.467	119.801	83.5066
**APD_{90} (ms)**	185.34	148.88	97.8698
**APD_{99} (ms)**	212.22	174.183	105.082

Table S1. Main parameters of AP waveforms. Diastolic potential (E_{diast}); peak amplitude of AP (PA) and action potential duration (APD) at different % of repolarization.
